# Leveraging Artificial Intelligence to Optimize Transcranial Direct Current Stimulation for Long COVID Management: A Forward-Looking Perspective

**DOI:** 10.3390/brainsci14080831

**Published:** 2024-08-19

**Authors:** Thorsten Rudroff, Oona Rainio, Riku Klén

**Affiliations:** Turku PET Centre, University of Turku, Turku University Hospital, 20520 Turku, Finland; oona.r.rainio@utu.fi (O.R.); riku.klen@utu.fi (R.K.)

**Keywords:** long COVID, brain stimulation, artificial intelligence, neuroimaging

## Abstract

Long COVID (Coronavirus disease), affecting millions globally, presents unprecedented challenges to healthcare systems due to its complex, multifaceted nature and the lack of effective treatments. This perspective review explores the potential of artificial intelligence (AI)-guided transcranial direct current stimulation (tDCS) as an innovative approach to address the urgent need for effective Long COVID management. The authors examine how AI could optimize tDCS protocols, enhance clinical trial design, and facilitate personalized treatment for the heterogeneous manifestations of Long COVID. Key areas discussed include AI-driven personalization of tDCS parameters based on individual patient characteristics and real-time symptom fluctuations, the use of machine learning for patient stratification, and the development of more sensitive outcome measures in clinical trials. This perspective addresses ethical considerations surrounding data privacy, algorithmic bias, and equitable access to AI-enhanced treatments. It also explores challenges and opportunities for implementing AI-guided tDCS across diverse healthcare settings globally. Future research directions are outlined, including the need for large-scale validation studies and investigations of long-term efficacy and safety. The authors argue that while AI-guided tDCS shows promise for addressing the complex nature of Long COVID, significant technical, ethical, and practical challenges remain. They emphasize the importance of interdisciplinary collaboration, patient-centered approaches, and a commitment to global health equity in realizing the potential of this technology. This perspective article provides a roadmap for researchers, clinicians, and policymakers involved in developing and implementing AI-guided neuromodulation therapies for Long COVID and potentially other neurological and psychiatric conditions.

## 1. Introduction

### AI-Guided Transcranial Direct Current Stimulation for Long COVID—A New Frontier in Neuromodulation

The emergence of Long COVID, a complex and debilitating condition affecting a significant proportion of individuals following severe acute respiratory syndrome coronavirus 2 (SARS-CoV-2) infection, has presented unprecedented challenges to healthcare systems worldwide. Long COVID is an often-debilitating illness that occurs in at least 10% of SARS-CoV-2 infections. More than 200 symptoms have been identified with impacts on multiple organ systems. At least 65 million individuals worldwide are estimated to have Long COVID, with cases increasing daily [1]. Characterized by a wide range of persistent symptoms including fatigue, cognitive dysfunction, and neurological disturbances, Long COVID has defied conventional treatment approaches and highlighted the need for innovative therapeutic strategies. In this context, the convergence of two cutting-edge fields—transcranial direct current stimulation (tDCS) and artificial intelligence (AI)—offers a promising avenue for addressing the multifaceted nature of Long COVID. The potential of AI in healthcare extends beyond Long COVID, with promising applications in various neurological conditions. For instance, in the field of migraines, AI has shown significant potential in diagnosis, prediction of attacks, and personalization of treatment. Torrente et al. [2] provided a comprehensive review of AI applications in migraines, highlighting its use in neuroimaging analysis, electronic diary data interpretation, and even in the development of novel therapeutic approaches [2]. Such advancements in high-prevalence neurological disorders provide a strong rationale for exploring AI applications in the context of Long COVID, particularly given the neurological symptoms often associated with the condition.

tDCS, a non-invasive neuromodulation technique, has shown potential in treating various neurological and psychiatric conditions. As demonstrated by Nitsche and Paulus [3], applying weak electrical currents (typically 1–2 mA) to specific areas of the brain can modulate neural excitability and potentially alleviate symptoms associated with Long COVID. Stagg and Nitsche [4] explain that the technique works by inducing polarity-specific effects: anodal stimulation generally increases cortical excitability, while cathodal stimulation typically decreases it. Polanía et al. [5] have shown that this modulation of neural activity can lead to both immediate and lasting changes in brain function and connectivity.

tDCS has demonstrated efficacy in conditions that share symptomatic overlap with Long COVID, such as chronic fatigue syndrome [6], cognitive impairment [7], and depression [8]. These findings suggest potential applicability to Long COVID, where neuroinflammation, altered brain connectivity, and disrupted neural networks may contribute to persistent symptoms, as described by Nalbandian et al. [9]. However, Wiethoff et al. [10] have highlighted that the heterogeneity of Long COVID presentations and the individual variability in response to tDCS present significant challenges in optimizing treatment protocols.

Current tDCS approaches often employ standardized protocols, which may be insufficient for addressing the complex and varied manifestations of Long COVID. This is where artificial intelligence enters the picture. AI, with its capacity for processing vast amounts of complex data and identifying subtle patterns, holds the promise of personalizing and optimizing tDCS treatments for Long COVID patients. By analyzing diverse datasets including clinical symptoms, neuroimaging data, and real-time physiological measurements, AI algorithms can potentially tailor tDCS parameters to individual patients, adapt treatments in real time, and predict long-term outcomes.

The integration of AI and tDCS in the context of Long COVID treatment is not merely a technological advancement; it represents a paradigm shift in how we approach complex, chronic conditions. This novel approach has the potential to address several key challenges in Long COVID management:

1. Personalization: Given the diverse manifestations of Long COVID, AI can help identify optimal tDCS protocols for individual patients based on their unique symptom profiles and physiological characteristics.
2. Real-time adaptation: AI algorithms can potentially adjust tDCS parameters in real time based on patient responses, allowing for dynamic, responsive treatment.
3. Predictive modeling: By analyzing large datasets, AI can help predict which patients are most likely to benefit from tDCS, optimizing resource allocation and improving patient outcomes.
4. Objective outcome measures: AI can assist in developing more sensitive and objective measures of treatment efficacy, crucial in a condition as complex and subjective as Long COVID.
5. Clinical trial optimization: AI-enhanced adaptive trial designs can potentially accelerate the process of identifying effective tDCS protocols for Long COVID.

However, Char et al. [11] warn that the integration of AI into medical treatments, particularly in the context of a novel condition like Long COVID, raises important ethical considerations. Issues of data privacy, algorithmic bias, equitable access to AI-enhanced treatments, and the need for transparency in AI decision-making processes must be carefully addressed. Furthermore, Wahl et al. [12] point out that the global implementation of AI-guided tDCS for Long COVID presents unique challenges and opportunities across different healthcare contexts. From high-income countries with advanced healthcare infrastructure to resource-limited settings, the successful deployment of this technology will require tailored approaches and innovative solutions.

This comprehensive perspective aims to explore the potential of AI-guided tDCS for Long COVID treatment, examining its promise, challenges, and future directions. We will delve into the technical aspects of AI-driven personalization and optimization of tDCS, discuss the ethical implications of this approach, outline key areas for future research, and consider strategies for global implementation.

The subsequent chapters will address the following key areas:

1. The promise of AI in addressing Long COVID challenges for tDCS.
2. AI-enhanced clinical trial design for tDCS in Long COVID.
3. Ethical considerations and challenges.
4. Future research directions.
5. Global implementation of AI-guided tDCS for Long COVID.

By comprehensively examining these aspects, we aim to provide a roadmap for researchers, clinicians, policymakers, and industry stakeholders involved in the development and implementation of AI-guided tDCS for Long COVID. As we stand at the intersection of neuroscience, artificial intelligence, and global health, the potential impact of this innovative approach extends beyond Long COVID, potentially paving the way for new paradigms in the treatment of complex neurological and psychiatric conditions.

In the face of the ongoing global challenge posed by Long COVID, the exploration of AI-guided tDCS represents a beacon of hope. While significant work remains unresolved, the potential benefits of this approach warrant rigorous investigation and thoughtful implementation. As we embark on this journey, it is crucial that we proceed with scientific rigor, ethical vigilance, and a steadfast commitment to improving the lives of those affected by Long COVID. Before exploring the potential of AI-guided tDCS for Long COVID treatment, it is crucial to understand the definition and complex pathophysiology of this condition, which informs our approach to developing targeted interventions.

## 2. Definition and Pathophysiology of Long COVID Syndrome Definition of Long COVID

Long COVID, also known as Post-Acute Sequelae of SARS-CoV-2 infection (PASC), is a complex multisystem disorder that persists or develops beyond 4 weeks of the initial SARS-CoV-2 infection [9]. The World Health Organization (WHO) has proposed the following clinical case definition [13]: “Post COVID-19 condition occurs in individuals with a history of probable or confirmed SARS-CoV-2 infection, usually 3 months from the onset of COVID-19 with symptoms that last for at least 2 months and cannot be explained by an alternative diagnosis”. Common symptoms include but are not limited to:

- Fatigue.
- Shortness of breath.
- Cognitive dysfunction (“brain fog”).
- Sleep disturbances.
- Persistent cough.
- Chest pain.
- Heart palpitations.
- Joint and muscle pain.
- Loss of taste or smell.
- Depression and anxiety.

It is important to note that symptoms can fluctuate or relapse over time, significantly impacting daily functioning and quality of life [14].

### Pathophysiological Origins of Long COVID

The exact pathophysiological mechanisms underlying Long COVID are still under investigation, but several key processes have been identified:

1. Persistent Inflammation: Evidence suggests ongoing systemic inflammation plays a crucial role in Long COVID. Elevated levels of pro-inflammatory cytokines and chemokines have been observed in Long COVID patients, indicating a dysregulated immune response [15].
2. Endothelial Dysfunction: SARS-CoV-2 can directly infect endothelial cells, leading to widespread endothelial dysfunction. This can result in microvascular damage and contribute to various symptoms, including cognitive impairment and fatigue [16].
3. Autoimmunity: Some studies have found evidence of autoantibodies in Long COVID patients, suggesting that autoimmune processes may contribute to ongoing symptoms [17].
4. Viral Persistence: There is evidence that SARS-CoV-2 viral reservoirs may persist in some patients, potentially driving ongoing inflammation and symptoms [18].
5. Mitochondrial Dysfunction: Impaired mitochondrial function has been observed in Long COVID patients, which could contribute to fatigue and exercise intolerance [18].
6. Autonomic Nervous System Dysregulation: Many Long COVID symptoms, such as tachycardia and orthostatic intolerance, may be attributed to dysfunction of the autonomic nervous system [19].
7. Microclots and Coagulation Abnormalities: Persistent microclots and hypercoagulability have been observed in some Long COVID patients, potentially contributing to tissue hypoxia and various symptoms [20].
8. Neuroinflammation: Brain imaging studies have shown evidence of neuroinflammation in Long COVID patients, which may underlie cognitive symptoms and fatigue [21].

## 3. Non-Invasive Brain Stimulation (NIBS) Techniques

Non-invasive brain stimulation (NIBS) encompasses various methods to modulate brain activity without surgical intervention. Common NIBS techniques include:

1. Transcranial Magnetic Stimulation (TMS).
2. Transcranial Direct Current Stimulation (tDCS).
3. Transcranial Alternating Current Stimulation (tACS).
4. Transcranial Random Noise Stimulation (tRNS).

These techniques have shown promise in treating various neurological and psychiatric conditions [22].

### 3.1. Transcranial Direct Current Stimulation (tDCS)

tDCS involves applying weak direct currents (typically 1–2 mA) to the scalp using electrodes, modulating neuronal excitability in targeted brain regions [3].

### 3.2. Methodology

tDCS methodology encompasses various aspects that influence its application and effects. Anodal stimulation generally increases cortical excitability, while cathodal stimulation typically decreases it [4]. Protocols can involve single or multiple sessions, with durations typically ranging from 10–30 min and current intensities usually between 1 and 2 mA [23]. The timing of stimulation relative to task performance is also important, with online stimulation applied during task performance and offline stimulation applied before [24]. Different montage types are used, including unilateral setups with one active electrode over the target area, and bilateral configurations with two active electrodes over different areas [25]. The choice of methodology depends on the specific therapeutic goals and the condition being treated.

### 3.3. Limitations and Challenges

Despite its potential, tDCS faces several limitations and challenges. Identifying the optimal stimulation sites for specific symptoms or conditions remains a significant challenge, as the association between stimulation location and therapeutic effect is complex [26]. Determining the most effective stimulation parameters, including intensity, duration, and frequency, for individual patients is also difficult due to the high degree of inter-individual variability in response to tDCS [27,28]. The long-term effects of tDCS and the optimal treatment schedules for maintaining therapeutic benefits are not fully established, which complicates the development of long-term treatment plans [29]. Additionally, while research has made significant strides, the precise neurophysiological mechanisms underlying tDCS effects are still being elucidated, which can make it challenging to predict and optimize outcomes [30].

These limitations highlight the potential benefit of using AI to optimize tDCS protocols for individual patients, particularly in complex conditions like Long COVID. AI could potentially help address the challenges of targeting, parameter optimization, and accounting for individual variability, leading to more personalized and effective tDCS interventions.

### 3.4. Relevance to tDCS and AI Applications

Understanding the complex and multifaceted nature of Long COVID’s pathophysiology is crucial for developing effective treatments, including AI-guided tDCS approaches. The diversity of underlying mechanisms contributes to the heterogeneity of Long COVID presentations, underscoring the potential value of personalized treatment approaches.

For instance, tDCS targeting the dorsolateral prefrontal cortex might be particularly beneficial for patients with predominant cognitive symptoms, while protocols focusing on pain-related areas might be more suitable for those with persistent musculoskeletal symptoms. AI algorithms could potentially help identify the most appropriate tDCS protocols based on individual symptom profiles and underlying pathophysiological patterns.

Moreover, the fluctuating nature of Long COVID symptoms highlights the potential benefit of adaptive treatment approaches, where AI could assist in real-time adjustment of tDCS parameters based on day-to-day symptom variations.

By grounding our approach in a clear understanding of Long COVID’s definition and pathophysiology, we can better tailor AI-guided tDCS interventions to address the specific challenges posed by this complex condition.

## 4. The Promise of AI in Addressing Long COVID Challenges for tDCS

The application of AI in healthcare has shown tremendous potential in recent years, particularly in its role in optimizing tDCS for Long COVID patients. This section explores how AI can be leveraged to personalize treatment and address the unique challenges presented by Long COVID in the context of tDCS therapy.

### 4.1. AI-Driven Personalization and Real-Time Optimization of tDCS for Long COVID

#### 4.1.1. Predictive Modeling for Responder Identification, Considering Long COVID-Specific Factors

AI-driven predictive modeling has the potential to significantly enhance the identification of potential tDCS responders among Long COVID patients. This approach can consider a wide range of factors specific to Long COVID, leading to more accurate predictions and improved treatment outcomes. Several machine learning algorithms have shown promise in this area.

Support Vector Machines (SVMs): Patel et al. [31] demonstrated the use of an SVM in predicting tDCS outcomes in depression. Their model considered demographic data, clinical symptoms, and neurophysiological markers. This approach could be adapted for Long COVID patients, incorporating factors such as the severity and duration of initial COVID-19 infection, specific Long COVID symptoms, and relevant biomarkers. The SVM algorithm’s ability to handle complex, high-dimensional data makes it particularly suitable for the multifaceted nature of Long COVID.

Random Forests: A study by Khadka et al. [32] utilized random forest algorithms to predict individualized electric fields in tDCS. This method could be especially useful in accounting for the neuroanatomical changes observed in some Long COVID patients. Random forests offer the advantage of handling both categorical and continuous variables, which is crucial given the diverse range of data types involved in Long COVID assessment. Furthermore, their ability to provide feature importance rankings could help identify the most critical factors influencing the tDCS response in Long COVID patients.

Deep Learning: Mansouri et al. [33] employed deep learning techniques to predict the cognitive enhancement effects of tDCS. Similar approaches could be developed for Long COVID, incorporating symptom profiles and neuroimaging data. Deep learning models, particularly convolutional neural networks (CNNs) and recurrent neural networks, could be used to analyze complex temporal patterns in Long COVID symptom progression and their association with the tDCS response. These models could potentially uncover subtle patterns that traditional statistical methods might miss. CNNs are typically used for image data, making them well-suited for analyzing medical imaging results. tDCS could potentially be combined with medical imaging techniques like positron emission tomography (PET) to evaluate treatment response. For instance, if Long COVID produces changes in glucose metabolism in areas like the hippocampus, PET images taken before and after tDCS treatment could be analyzed using CNNs to assess the effectiveness of the intervention and detect subtle changes in brain metabolism that may correlate with symptom improvement.

Feature selection is a critical aspect of developing effective AI models for tDCS response prediction in Long COVID patients. Relevant features to consider include COVID-19 severity and duration, specific Long COVID symptoms and their severity, neuroimaging data (structural and functional MRI, DTI), genetic markers, inflammatory biomarkers, and cognitive performance metrics. The integration of these diverse data types presents both a challenge and an opportunity for AI-driven approaches. Table 1 overviews various AI techniques and how they might be applied specifically to optimizing tDCS for Long COVID treatment.

Debnath et al. [34] developed a multi-modal AI approach combining EEG, fMRI, and clinical data to predict treatment response in major depressive disorder. A similar strategy could be employed for Long COVID, integrating neurophysiological data with symptom profiles and biomarkers. This multi-modal approach could provide a more comprehensive understanding of the factors influencing tDCS response in Long COVID patients.

Given the global nature of the COVID-19 pandemic, federated learning approaches could be particularly valuable. This AI technique allows for the development of predictive models using data from multiple institutions without compromising patient privacy. Silva et al. [35] demonstrated the potential of federated learning in healthcare, which could be applied to tDCS research in Long COVID across different countries and healthcare systems. This approach could facilitate large-scale, international collaborations while addressing data privacy concerns and regulatory differences across jurisdictions (Table 1).

#### 4.1.2. Real-Time Adjustment of Stimulation Parameters Based on Symptom Fluctuations

The dynamic nature of Long COVID symptoms necessitates adaptive tDCS protocols. AI can play a crucial role in enabling real-time adjustments to stimulation parameters based on symptom fluctuations. Several approaches show promise in this area.

Closed-loop Systems: AI-driven closed-loop systems can continuously monitor patient data and adjust tDCS parameters in real time. While such systems are still in the early stages of development for tDCS, they have shown promise in other neuromodulation techniques. Bouthour et al. [36] demonstrated the potential of adaptive deep brain stimulation based on real-time electroencephalogram (EEG) analysis. Similar principles could be applied to tDCS in Long COVID, adjusting stimulation based on ongoing brain activity. This could involve the use of machine learning algorithms to identify EEG signatures associated with symptom improvement or deterioration, triggering automatic adjustments to tDCS parameters.

Physiological Feedback: AI algorithms could integrate data from wearable devices monitoring heart rate variability, skin conductance, or other physiological markers as proxies for symptom fluctuations, adjusting tDCS parameters accordingly. This approach could leverage advances in the Internet of Things and edge computing to process physiological data in real-time, enabling rapid responses to changes in patient state.

Reinforcement Learning: Reinforcement learning algorithms could be employed to optimize tDCS parameters over time based on patient outcomes. Lorenz et al. [37] used Q-learning to optimize deep brain stimulation parameters in Parkinson’s disease. A similar approach could be adapted for tDCS in Long COVID, learning optimal stimulation patterns based on symptom improvements. This would allow the system to continuously refine its stimulation strategy, potentially leading to increasingly effective treatments over time.

Time Series Analysis: AI techniques for time series analysis can be valuable in predicting symptom fluctuations and proactively adjusting tDCS protocols. Long Short-Term Memory (LSTM) networks have shown efficacy in predicting time series data in healthcare. Lipton et al. [38] demonstrated their use in predicting diagnoses in clinical time series, which could be adapted to predict Long COVID symptom fluctuations. These models could potentially forecast symptom trajectories, allowing for preemptive adjustments to tDCS parameters.

Natural Language Processing (NLP): NLP techniques can be employed to analyze patient-reported outcomes and adjust tDCS parameters accordingly. Sentiment analysis of patient feedback could provide insights into subjective experiences with tDCS, informing parameter adjustments. Topic modeling techniques could be used to identify emerging symptom patterns from patient reports, allowing for targeted adjustment of tDCS protocols. This approach could help capture the nuanced and often subjective nature of Long COVID symptoms.

Neuroimaging techniques such as positron emission tomography (PET) and magnetic resonance imaging (MRI) can provide valuable data for personalizing tDCS protocols. PET imaging could be used to assess metabolic activity and inflammation in specific brain regions affected by Long COVID, while functional and structural MRI could reveal altered connectivity patterns and structural changes. AI algorithms could analyze these neuroimaging datasets to identify optimal stimulation targets and parameters for individual patients. For example, machine learning models could be trained to correlate patterns of brain activity or structural changes seen on neuroimaging with symptom severity and tDCS response, allowing for more precise targeting of stimulation.

In conclusion, AI holds immense promise in addressing the challenges of tDCS application in Long COVID patients. By leveraging advanced machine learning techniques for predictive modeling and real-time parameter adjustment, AI can enable truly personalized and adaptive tDCS protocols. However, it is crucial to note that while these AI applications show great potential, many are still in the early stages of development and require rigorous clinical validation before widespread implementation. Future research should focus on developing robust, interpretable AI models that can be seamlessly integrated into clinical practice, always prioritizing patient safety and ethical considerations.

## 5. AI-Enhanced Clinical Trial Design for tDCS in Long COVID

**Figure 1:**
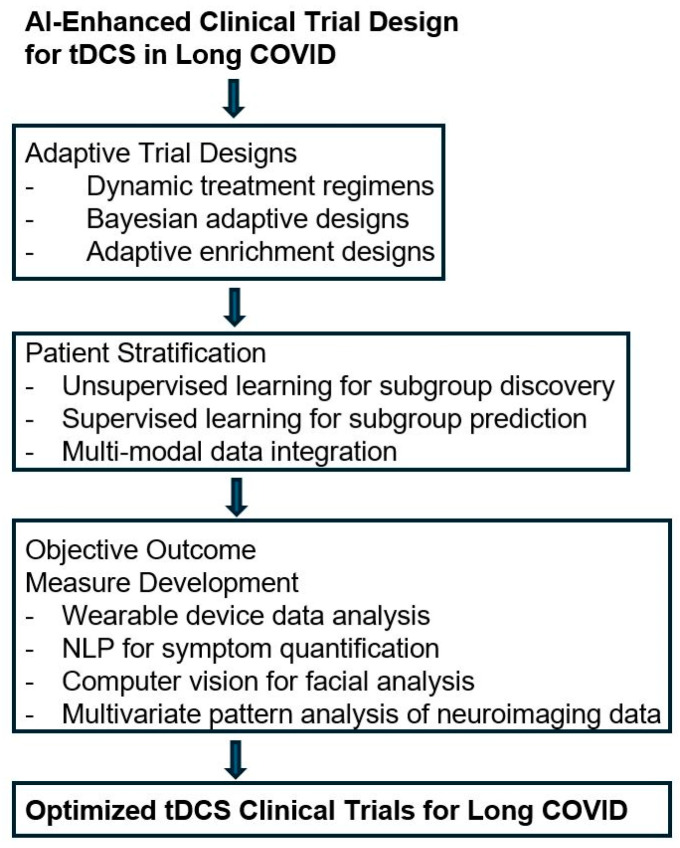
AI-enhanced clinical trial design for tDCS in Long COVID.

The application of Artificial Intelligence (AI) in clinical trial design offers significant potential to optimize the evaluation of transcranial direct current stimulation (tDCS) for Long COVID patients. This section explores how AI can enhance adaptive trial designs, patient stratification, and outcome measure development in this context. As illustrated in Figure 1, AI-enhanced clinical trial design for tDCS in Long COVID incorporates three key components: adaptive trial designs, patient stratification, and objective outcome measure development. These AI-driven approaches aim to optimize the efficiency, precision, and clinical relevance of tDCS trials for Long COVID treatment. The flowchart demonstrates how these components work together to create a more responsive and personalized clinical trial framework, potentially leading to more effective evaluation of tDCS interventions for Long COVID patients.

The flowchart begins with the overall concept and flows through three main stages:

1. Adaptive trial designs: This stage incorporates AI-driven approaches to make trials more flexible and responsive to emerging data. It includes dynamic treatment regimens, Bayesian adaptive designs, and adaptive enrichment designs.
2. Patient stratification: This stage uses AI techniques to identify more homogeneous subgroups within the heterogeneous Long COVID population. It includes unsupervised learning for subgroup discovery, supervised learning for subgroup prediction, and multi-modal data integration.
3. Objective outcome measure development: This final stage leverages AI to develop more sensitive and objective measures of treatment efficacy. It includes wearable device data analysis, natural language processing for symptom quantification, computer vision for facial analysis, and multivariate pattern analysis of neuroimaging data.

The flowchart concludes with the end goal of optimized tDCS clinical trials for Long COVID. This structure illustrates how AI can enhance various aspects of clinical trial design, potentially leading to more efficient, precise, and clinically relevant studies of tDCS for Long COVID treatment.

### 5.1. Adaptive Trial Designs Using Machine Learning

The complexity and variability of Long COVID symptoms present unique challenges for clinical trial design. Traditional fixed trial designs may be insufficient to capture the dynamic nature of the condition and the potential variability in tDCS response. AI-driven adaptive trial designs offer a promising solution to these challenges.

#### Incorporating Long COVID Symptom Fluctuations into Trial Protocols

Long COVID is characterized by fluctuating symptoms that can vary significantly over time and between patients. This variability necessitates flexible trial designs that can adapt to changing patient conditions. Several AI-driven approaches show promise in this area.

Dynamic treatment regimens: Chakraborty and Murphy [39] demonstrated the use of Q-learning, a type of reinforcement learning algorithm, for optimizing dynamic treatment regimes. The authors applied this method to simulate data for a chronic psychiatric disorder, showing how it could personalize treatment sequences. This approach could be adapted for tDCS in Long COVID by optimizing stimulation parameters and timing based on individual patient responses. For example, an AI system could learn to adjust tDCS protocols based on daily symptom reports, cognitive test results, and physiological measurements, creating a personalized treatment trajectory for each patient.

Bayesian adaptive designs: Berry et al. [40] provided a comprehensive review of Bayesian adaptive designs in clinical trials. These designs can incorporate accumulating data to adjust various trial parameters, such as sample size, treatment allocation, or dose selection. The authors highlighted examples in oncology and cardiovascular medicine, suggesting potential applications in complex conditions like Long COVID. In the context of tDCS for Long COVID, a Bayesian adaptive design could allow for the continuous reassessment of the most effective stimulation parameters as data accumulates throughout the trial. This could lead to more efficient identification of optimal treatment protocols and potentially shorter trial durations.

Adaptive enrichment designs: Simon and Simon [41] proposed an adaptive enrichment design that allows researchers to modify enrollment criteria based on interim analyses. The authors demonstrated how this approach could increase the power to detect treatment effects in responsive subgroups. For tDCS in Long COVID, this could help identify and focus on patient subgroups most likely to benefit from the treatment. For instance, if interim analyses suggest that patients with specific cognitive symptoms respond particularly well to tDCS, the trial could adapt to enroll more patients with these characteristics, increasing the likelihood of detecting significant treatment effects.

These adaptive trial designs, enhanced by AI, offer several advantages for studying tDCS in Long COVID:

1. Efficiency: By adapting to emerging data, these designs can potentially reach conclusions faster than traditional fixed designs.
2. Ethical considerations: Adaptive designs can potentially expose fewer patients to ineffective treatments by adjusting allocation based on accumulating evidence.
3. Personalization: These approaches align well with the goal of personalized medicine, allowing for the identification of patient subgroups most likely to benefit from tDCS.
4. Flexibility: Adaptive designs can accommodate the uncertainty inherent in studying a novel condition like Long COVID, allowing for adjustments as our understanding of the disease evolves.

### 5.2. Patient Stratification and Targeted Recruitment

The heterogeneous nature of Long COVID presents challenges for patient selection and treatment evaluation in clinical trials. AI-driven approaches to patient stratification can help identify more homogeneous subgroups, potentially leading to more targeted and effective treatments.

#### Identifying Homogeneous Subgroups within the Heterogeneous Long COVID Population

Several AI techniques have shown promise for patient stratification in Long COVID:

Unsupervised learning for subgroup discovery: Sudre et al. [42] used k-means clustering, an unsupervised learning algorithm, to identify symptom clusters in Long COVID patients. The researchers analyzed data from 4182 individuals, identifying six distinct symptom clusters. This approach could be extended to include tDCS response data, potentially revealing subgroups more likely to benefit from the treatment. For example, an unsupervised learning algorithm could identify clusters of patients based on a combination of symptoms, biomarkers, and initial tDCS responses, allowing for more targeted treatment strategies.

Supervised learning for subgroup prediction: Qian et al. [43] demonstrated the use of random forests, a supervised learning algorithm, for patient stratification in Alzheimer’s disease. The authors used genetic, cognitive, and neuroimaging data to predict disease progression. A similar approach could be adapted for Long COVID, using relevant features to predict which patients might respond best to tDCS. This could involve training a model on data from initial tDCS responders and non-responders, and then using this model to predict likely responders among new patients.

Multi-modal data integration: Wang et al. [44] used tensor factorization, a technique for analyzing multi-dimensional data, to integrate various types of neuroimaging data in Alzheimer’s disease. This method could be applied to Long COVID by incorporating tDCS response data alongside other clinical and biological measures, potentially revealing more nuanced patient subgroups. For instance, a tensor factorization approach could simultaneously analyze symptom profiles, neuroimaging data, inflammatory markers, and tDCS response patterns, uncovering complex associations that might not be apparent when examining each data type in isolation.

These AI-driven stratification approaches offer several benefits for tDCS trials in Long COVID:

1. Improved trial efficiency: By identifying more homogeneous patient subgroups, these methods could reduce noise in treatment effect estimates, potentially allowing for smaller sample sizes.
2. Personalized treatment strategies: Stratification could guide the development of tailored tDCS protocols for different patient subgroups.
3. Mechanistic insights: Identifying distinct patient subgroups could provide insights into the underlying mechanisms of both Long COVID and tDCS effects.
4. Improved patient selection: AI-driven stratification could help identify the patients most likely to benefit from tDCS, improving the risk–benefit ratio for trial participants.

### 5.3. Objective Outcome Measure Development Using AI

The diverse and often subjective nature of Long COVID symptoms presents challenges for outcome assessment in clinical trials. AI techniques can help develop more objective and sensitive measures to capture subtle improvements in Long COVID symptoms following tDCS treatment.

#### Developing Sensitive Measures to Capture Subtle Improvements in Long COVID Symptoms

Several AI approaches show promise in this area:

Wearable device data analysis: Manta et al. [45] used deep learning to analyze accelerometer data for assessing fatigue in multiple sclerosis patients. The researchers developed a convolutional neural network model that could distinguish between fatigued and non-fatigued states. This approach could be adapted to assess fatigue and other physical symptoms in Long COVID patients undergoing tDCS treatment. For example, AI models could analyze data from wrist-worn accelerometers to quantify changes in activity levels and sleep patterns as objective measures of fatigue improvement.

Natural language processing (NLP) for symptom quantification: Dreisbach et al. [46] demonstrated the use of NLP techniques to analyze patient-reported symptoms in electronic health records. The authors developed and validated an NLP system for extracting symptoms related to peripheral artery disease. Similar methods could be applied to analyze symptom diaries or other text-based reports from Long COVID patients in tDCS trials. This could involve using sentiment analysis to quantify changes in symptom severity over time, or topic modeling to track shifts in the predominant symptoms reported by patients.

Computer vision for facial analysis: Bandini et al. [47] used computer vision techniques to assess facial expressiveness in Parkinson’s disease patients. The researchers developed an automated system to quantify facial movements during standard neurological examinations. A similar approach could potentially be used to assess neurological symptoms or overall well-being in Long COVID patients undergoing tDCS. For instance, AI-driven facial analysis could quantify changes in facial expressions associated with mood or cognitive effort during standardized tasks.

Multivariate pattern analysis of neuroimaging data: Woo et al. [48] provided a comprehensive review of various machine learning approaches for developing neuroimaging-based biomarkers. The authors covered techniques such as support vector machines, random forests, and deep learning, explaining their applications in neurological and psychiatric disorders. These methods could be applied to assess the effects of tDCS on brain structure and function in Long COVID patients. For example, machine learning models could be trained to identify patterns of brain activation or connectivity associated with symptom improvement following tDCS treatment.

Neuroimaging-based outcome measures, analyzed using AI, could provide objective markers of tDCS efficacy. For instance, changes in functional connectivity patterns observed on functional MRI (fMRI), or alterations in metabolic activity seen on PET, could be quantified using machine learning algorithms. These AI-derived neuroimaging markers could serve as more sensitive and objective measures of treatment response compared to subjective symptom reports alone.

These AI-driven approaches to outcome measure development offer several advantages for tDCS trials in Long COVID:

1. Objectivity: AI-based measures can provide more objective assessments of symptoms that are typically subjective, potentially reducing bias and improving reliability.
2. Sensitivity: AI techniques can potentially detect subtle changes in patient status that might be missed by traditional clinical assessments.
3. Continuous monitoring: Many of these approaches allow for continuous or frequent assessment, providing a more detailed picture of symptom trajectories.
4. Multi-dimensional assessment: AI can integrate data from multiple sources, providing a more comprehensive view of patient status.
5. Scalability: Once developed, many AI-based assessments can be deployed at scale, potentially allowing for larger and more efficient trials.

In conclusion, AI offers powerful tools for enhancing clinical trial design for tDCS in Long COVID patients. From adaptive trial designs to patient stratification and novel outcome measures, AI techniques have the potential to increase the efficiency, precision, and clinical relevance of these trials. However, it is important to note that these approaches are still evolving, and their implementation will require careful validation and consideration of ethical implications. Future research should focus on rigorously evaluating these AI-enhanced trial designs and outcome measures in the specific context of tDCS for Long COVID.

## 6. Ethical Considerations and Challenges in AI-Guided tDCS for Long COVID Treatment

The application of AI-guided transcranial direct current stimulation (tDCS) for Long COVID treatment presents a complex landscape of ethical considerations, encompassing issues of data privacy, equitable access, transparency, and patient safety. These challenges are further complicated by the evolving nature of both AI technology and our understanding of Long COVID. In this context, the European Union’s AI Act provides a crucial framework for addressing these ethical concerns and guiding the responsible development and deployment of AI in healthcare.

### 6.1. Data Privacy and Security

Data privacy and security remain paramount concerns in the development of AI-guided tDCS systems. The implementation of privacy-preserving techniques such as federated learning and differential privacy [49] is essential to protect patient information while enabling the collaborative development of robust AI models. Effective anonymization and de-identification methods must be employed to maintain data utility while safeguarding individual privacy [50]. The establishment of secure data-sharing frameworks [51] and clear consent processes with well-defined data ownership policies [52] are crucial steps in building trust and ensuring ethical data-handling practices.

### 6.2. Equitable Access and Algorithmic Bias

Ensuring equitable access to AI-enhanced treatments is a critical ethical imperative. This involves addressing the digital divide in healthcare [53] and mitigating algorithmic bias through the use of diverse training data [54]. Careful consideration of resource allocation and global health equity is necessary to prevent the exacerbation of existing healthcare disparities [10,12]. The AI Act’s emphasis on high-risk AI systems could potentially impact the accessibility of AI-guided tDCS systems, as stringent regulatory requirements may increase development costs and time-to-market. However, these measures also serve to ensure the safety and reliability of such systems, potentially increasing trust and adoption in the long term.

### 6.3. Transparency and Explainability

The “black box” problem in healthcare AI poses significant challenges to transparency and explainability in clinical decision-making [55]. Implementing explainable AI techniques [56] is crucial for building trust among patients and healthcare providers. The AI Act’s requirements for high-risk AI systems, including the need for human oversight and detailed documentation, align with these goals and could drive the development of more transparent and interpretable AI-guided tDCS systems.

### 6.4. Clinical Validation and Regulatory Frameworks

Rigorous clinical validation processes are essential for ensuring the safety and efficacy of AI-guided tDCS systems [57]. The AI Act’s risk-based approach and emphasis on high-risk AI systems in healthcare could significantly impact the development and deployment of AI-guided tDCS systems. These systems are likely to be classified as high-risk medical devices, subject to stringent requirements including risk management, data governance, and ongoing monitoring. While these regulations may present challenges in terms of development time and cost, they also provide a clear framework for ensuring patient safety and system reliability.

### 6.5. Long COVID-Specific Considerations

The unique challenges presented by Long COVID necessitate specific ethical considerations. These include developing fair resource allocation strategies in strained healthcare systems [58] and addressing informed consent challenges in the context of rapidly evolving technologies [59]. Managing patient expectations in a high-hope context is crucial [60], particularly given the novelty of both Long COVID and AI-guided tDCS treatments. The establishment of robust long-term data governance frameworks [61] is essential for tracking outcomes and refining treatment approaches over time.

### 6.6. AI Governance and the EU AI Act

The EU AI Act provides a comprehensive framework for addressing many of these ethical challenges [62]. Its risk-based classification system, ranging from unacceptable risk (prohibited) to minimal risk (unregulated), offers a nuanced approach to AI governance. For AI-guided tDCS systems, which are likely to fall under the high-risk category, the Act mandates specific requirements such as risk management, data governance, and human oversight.

The Act’s focus on providers (developers) of high-risk AI systems, with additional responsibilities for deployers, ensures accountability throughout the AI lifecycle. The specific requirements for General Purpose AI (GPAI) models, including additional obligations for those presenting systemic risks, may impact the development of more advanced AI systems for tDCS guidance. The prohibition of certain AI applications, such as those using manipulative techniques or social scoring, underscores the Act’s commitment to protecting fundamental rights. While these prohibitions may not directly affect AI-guided tDCS systems, they set an important ethical standard for AI development in healthcare.

The establishment of an AI Office to oversee implementation and compliance provides a centralized mechanism for addressing ethical concerns and ensuring adherence to the Act’s requirements. The phased implementation timeline, ranging from 6 to 36 months, allows for a gradual adaptation to these new regulatory requirements.

### 6.7. Conclusions

As our understanding of Long COVID and the capabilities of AI-guided tDCS evolve, these ethical considerations must be continually addressed and refined. The EU AI Act provides a valuable framework for navigating these challenges, but it must be complemented by an ongoing dialogue between researchers, clinicians, ethicists, policymakers, and patients. By proactively engaging with these ethical considerations and regulatory requirements, we can work towards a more equitable, transparent, and patient-centered approach to AI-guided tDCS for Long COVID treatment. This approach not only ensures compliance with legal standards but also builds trust and acceptance among patients and healthcare providers, ultimately leading to more effective and ethically sound treatment options for those affected by Long COVID.

## 7. Future Research Directions

To advance the field of AI-guided tDCS for Long COVID treatment, several key research questions need to be addressed. This section outlines critical areas for future investigation, highlighting the interdisciplinary nature of this research and the need for robust, patient-centered approaches.

As summarized in Table 2, future research directions for AI-guided transcranial direct current stimulation (tDCS) in Long COVID treatment span multiple domains. This table outlines eleven key research areas, their primary objectives, and potential approaches for investigation. These areas range from optimizing tDCS parameters and personalizing treatments to exploring long-term efficacy, integrating with other therapies, and addressing ethical and regulatory challenges. This comprehensive research agenda aims to advance the development and implementation of AI-guided tDCS as a potential treatment modality for Long COVID, emphasizing the need for interdisciplinary collaboration and patient-centered approaches.

### 7.1. Optimal tDCS Parameters for Long COVID

A fundamental question in this field is determining the most effective tDCS montages and stimulation parameters (e.g., intensity, duration, and frequency) for different Long COVID symptoms. This question is complicated by the heterogeneous nature of Long COVID, which may necessitate different optimal parameters for different patient subgroups.

To address this, large-scale, randomized controlled trials comparing different tDCS protocols are needed. These trials should incorporate AI analysis of patient characteristics and outcomes to identify optimal parameters for specific subgroups. For example, a trial might compare several tDCS montages across a diverse Long COVID patient population, using machine learning algorithms to analyze the relationship between patient characteristics (e.g., age, symptom profile, and duration of illness) and treatment response.

Hanlon et al. [63] demonstrated the potential of such an approach in their study of tDCS for alcohol use disorder. The authors used machine learning to identify patient characteristics predictive of treatment response, which could inform more personalized treatment approaches. A similar methodology could be applied to Long COVID, potentially revealing how factors such as the severity of initial COVID-19 infection or the presence of specific neurological symptoms might influence optimal tDCS parameters.

### 7.2. AI-Driven Personalization of tDCS

Building on the identification of optimal parameters, a key research direction is the development of AI algorithms that can accurately predict individual patient responses to tDCS based on clinical, demographic, and neurophysiological data. Furthermore, investigating how AI can be used to dynamically adjust tDCS parameters in real time based on patient responses is crucial for truly personalized treatment.

To pursue this, researchers should develop and validate machine learning models using large datasets of patient characteristics and tDCS outcomes. These models could then be implemented in closed-loop tDCS systems with AI-driven parameter adjustment, and their efficacy compared to standard protocols.

Lorenz et al. [37] demonstrated the potential of this approach in their work on adaptive deep brain stimulation for Parkinson’s disease. The authors used a reinforcement learning algorithm to optimize stimulation parameters in real time based on patient symptoms. A similar approach could be adapted for tDCS in Long COVID, potentially allowing for the dynamic adjustment of stimulation parameters based on fluctuating symptoms.

### 7.3. Long-Term Efficacy and Safety

Given the chronic nature of Long COVID, understanding the long-term effects of AI-guided tDCS on symptoms is crucial. Additionally, investigating any cumulative effects or safety concerns with repeated tDCS sessions over extended periods is essential for ensuring patient safety.

To address these questions, longitudinal studies following patients for 12–24 months post-treatment are needed. These studies should use AI to analyze long-term outcome patterns and identify potential safety signals. For example, machine learning algorithms could be employed to detect subtle patterns in symptom trajectories or to identify early indicators of adverse effects.

Brunoni et al. [64] conducted a long-term follow-up study of tDCS for depression, demonstrating the feasibility and importance of such longitudinal investigations. A similar approach, enhanced by AI analysis, could provide crucial insights into the long-term efficacy and safety of tDCS for Long COVID.

### 7.4. Integration with Other Treatments

Understanding how AI-guided tDCS interacts with other Long COVID treatments (e.g., medications, cognitive rehabilitation, and physical therapy) is another important area for future research. Additionally, investigating whether AI can optimize the timing and combination of tDCS with other interventions could lead to more comprehensive and effective treatment strategies.

To explore this, factorial studies combining tDCS with other treatments should be designed, using AI to analyze interaction effects and optimize treatment combinations. For instance, a study might compare AI-guided tDCS alone, cognitive rehabilitation alone, and a combination of the two, with AI algorithms analyzing the data to identify optimal treatment sequences or combinations for different patient subgroups.

Mohr et al. [65] demonstrated the potential of such combination approaches in their study on internet-based cognitive behavioral therapy combined with tDCS for depression. Applying similar methodologies, enhanced by AI, to Long COVID could lead to more integrated and effective treatment strategies.

### 7.5. Neuroplasticity and Mechanism of Action

Investigating how AI-guided tDCS affects brain plasticity and connectivity in Long COVID patients is crucial for understanding its mechanism of action. Furthermore, exploring whether AI can predict and optimize neuroplastic changes induced by tDCS could lead to more targeted and effective treatments.

To address these questions, studies combining tDCS with neuroimaging (e.g., fMRI, EEG) and using AI to analyze changes in brain structure and function, correlating these with symptom improvements, are needed. For example, machine learning algorithms could be used to identify patterns of functional connectivity changes associated with symptom improvement following tDCS treatment.

Cavaliere et al. [66] used machine learning to analyze fMRI data and predict cognitive training outcomes in patients with traumatic brain injury. A similar approach, applied to tDCS in Long COVID, could provide valuable insights into the neural mechanisms underlying treatment effects and potentially guide more targeted interventions.

### 7.6. Combining tDCS with Neuroimaging

Further research combining tDCS with advanced neuroimaging is crucial for elucidating the mechanisms of action in Long COVID treatment. Multimodal imaging approaches, integrating PET, fMRI, and EEG data, could provide a comprehensive view of how tDCS modulates brain activity and connectivity in Long COVID patients. AI techniques such as deep learning could be applied to these complex, multimodal datasets to uncover subtle patterns of brain changes associated with symptom improvement. This could lead to the development of imaging-based biomarkers for predicting and monitoring tDCS response. Table 3 displays neuroimaging modalities with AI-guided tDCS.

### 7.7. Patient-Reported Outcomes and Quality of Life

Incorporating patient-reported outcomes and quality-of-life measures into AI models for optimizing tDCS protocols is essential for ensuring that treatments are meaningfully improving patients’ lives. Identifying the most relevant patient-centered outcomes for assessing AI-guided tDCS efficacy in Long COVID is a crucial step in this process.

To achieve this, researchers should develop and validate AI models that integrate standardized quality-of-life measures and free-text patient narratives to guide tDCS optimization. Natural language processing techniques could be employed to analyze patient diaries or interview transcripts, extracting meaningful insights about treatment effects that may not be captured by standard clinical measures.

Sidey-Gibbons and Sidey-Gibbons [67] demonstrated the potential of machine learning for analyzing patient-reported outcome measures in their review of applications in quality-of-life research. Applying these techniques to Long COVID could provide a more comprehensive understanding of treatment effects and guide more patient-centered approaches to tDCS optimization.

### 7.8. Home-Based AI-Guided tDCS

Investigating the feasibility and safety of home-based, AI-guided tDCS for Long COVID patients is an important area for future research. This includes exploring how AI can ensure proper electrode placement and stimulation delivery in home settings, which is crucial for treatment efficacy and safety.

To address these questions, researchers should conduct feasibility studies of home-based tDCS systems with AI monitoring, comparing outcomes and safety profiles with clinic-based treatments. These studies should consider factors such as patient adherence, ease of use, and the reliability of remote monitoring systems.

Charvet et al. [68] demonstrated the feasibility of remotely supervised tDCS in multiple sclerosis patients, providing a model that could be adapted and enhanced with AI for Long COVID treatment. Future studies could build on this work by incorporating AI-driven adaptive protocols and real-time monitoring of treatment parameters and patient responses.

### 7.9. Health Economic Analysis

Understanding the cost-effectiveness of AI-guided tDCS compared to standard care for Long COVID is crucial for informing healthcare policy and resource allocation. Additionally, investigating how the integration of AI impacts the overall cost and accessibility of tDCS treatment is important for ensuring equitable access to care.

To explore these questions, researchers should perform health economic modeling studies, incorporating data on treatment efficacy, resource utilization, and quality of life improvements. These models should consider both direct medical costs and indirect costs such as lost productivity due to Long COVID symptoms.

Ghosh et al. [69] conducted a cost-effectiveness analysis of tDCS for depression, providing a methodological framework that could be adapted for Long COVID. Future studies could extend this approach by incorporating the potential cost savings and efficiency gains offered by AI-guided personalization of treatment.

### 7.10. Ethical and Regulatory Frameworks

Developing comprehensive ethical guidelines for the responsible development and implementation of AI-guided tDCS for Long COVID is a critical area for future research. This includes exploring how regulatory frameworks can be adapted to effectively evaluate and approve AI-driven neuromodulation technologies.

To address these questions, multidisciplinary studies involving ethicists, legal experts, clinicians, and patients should be conducted to develop comprehensive ethical guidelines and propose regulatory frameworks. These studies should consider issues such as data privacy, algorithmic bias, patient autonomy, and the unique challenges posed by adaptive AI systems.

Gerke et al. [70] examined the regulatory challenges of AI in healthcare, providing insights that could be applied to the specific context of AI-guided tDCS for Long COVID. Future research should build on this work to develop tailored regulatory approaches that balance innovation with patient safety and ethical considerations.

### 7.11. Generalizability and Health Disparities

Investigating the generalizability of AI models developed for tDCS in Long COVID across different populations and healthcare settings is crucial for ensuring equitable and effective treatment. Additionally, exploring whether AI-guided tDCS can help address health disparities in Long COVID treatment is an important area for future research.

To pursue these questions, multi-center, international studies should be conducted to validate AI models across diverse populations. Targeted studies in underserved communities should be implemented to assess the impact of AI-guided tDCS on health equity.

Veinot et al. [71] proposed a framework for addressing health disparities in AI-based health interventions, which could be applied to the development and implementation of AI-guided tDCS for Long COVID. Future research should build on this work to ensure that AI models are developed and validated using diverse, representative datasets and that implementation strategies are tailored to the needs of different communities.

### 7.12. Conclusions and Synthesis

The research agenda outlined above highlights the interdisciplinary nature of AI-guided tDCS for Long COVID treatment and the need for a patient-centered approach. By addressing these key research questions, the field can move towards more effective, personalized, and ethically sound applications of this promising technology.

Several overarching themes emerge from this research agenda:

1. Personalization: Many of the proposed research directions focus on leveraging AI to tailor tDCS treatment to individual patients, reflecting the heterogeneous nature of Long COVID.
2. Integration: There is a clear need to integrate AI-guided tDCS with other treatment modalities and to consider its implementation within broader healthcare systems.
3. Long-term perspectives: Given the chronic nature of Long COVID, understanding the long-term effects and safety of AI-guided tDCS is crucial.
4. Ethical considerations: Ethical issues are woven throughout the research agenda, reflecting the need to balance innovation with patient safety and equitable access to care.
5. Real-world implementation: Many of the proposed studies focus on translating AI-guided tDCS from controlled research settings to real-world clinical practice and home-based applications.

To effectively pursue this research agenda, collaboration across disciplines will be essential. This includes not only neuroscientists, AI researchers, and clinicians, but also ethicists, health economists, and patient advocates. Furthermore, engaging patients as active partners in the research process will be crucial for ensuring that AI-guided tDCS development aligns with the needs and preferences of those living with Long COVID.

As this field evolves, it will be important to regularly reassess and update the research agenda in light of new findings and emerging challenges. By maintaining a commitment to rigorous scientific inquiry, ethical practice, and patient-centered care, the development of AI-guided tDCS has the potential to significantly improve the lives of those affected by Long COVID.

## 8. Current Empirical Evidence for AI-Guided tDCS in Long COVID Treatment

While the potential of AI-guided tDCS for Long COVID treatment is promising, it is important to acknowledge that much of the discussion in this article remains theoretical at this stage. The novelty of both Long COVID and the application of AI to tDCS means that robust empirical data specifically addressing this combination are currently limited. However, we can draw insights from related areas of research that provide a foundation for our hypotheses.

### 8.1. tDCS in Post-Viral Fatigue Syndromes

Although not specifically focused on Long COVID, studies have shown potential benefits of tDCS in treating symptoms similar to those experienced in Long COVID:

Ferrucci et al. [72] conducted a randomized, sham-controlled study on 23 patients with chronic fatigue syndrome, finding that anodal tDCS over the left dorsolateral prefrontal cortex significantly reduced fatigue levels [72].

A case series by Tecchio et al. [6] demonstrated that personalized tDCS protocols targeting the somatosensory areas could alleviate fatigue in multiple sclerosis patients [6].

### 8.2. AI in tDCS Optimization

While not yet applied to Long COVID specifically, AI has shown promise in optimizing tDCS for other conditions:

Lorenz et al. [73] used Bayesian optimization, a form of AI, to personalize tDCS montages for enhancing working memory, demonstrating superior results compared to standard approaches [73].

A study by Csifcsák et al. [74] employed machine learning algorithms to predict individual responses to tDCS in a cognitive task, highlighting the potential of AI for personalized tDCS protocols [74].

### 8.3. AI in Long COVID Research

Although not specific to tDCS, AI has been utilized in Long COVID research.

Sudre et al. [42] used machine learning to analyze symptom clusters in Long COVID patients, identifying six distinct phenotypes [42].

An et al. [75] developed a deep learning model to predict Long COVID based on early symptoms and patient characteristics, achieving high accuracy [75].

### 8.4. Gaps and Future Directions

While these studies provide a basis for our hypotheses, several key gaps remain:

1. Direct empirical evidence of AI-guided tDCS efficacy in Long COVID is lacking.
2. Long-term effects and safety profiles of AI-optimized tDCS protocols need investigation.
3. The impact of AI-guided tDCS on diverse Long COVID symptoms beyond fatigue requires study.
4. Large-scale, randomized controlled trials are needed to validate the proposed approaches.

To address these gaps, we propose the following priorities for future research:

1. Conduct pilot studies specifically testing AI-guided tDCS protocols in Long COVID patients.
2. Develop and validate AI models for predicting individual responses to tDCS in Long COVID.
3. Perform longitudinal studies to assess long-term efficacy and safety.
4. Investigate the potential of AI-guided tDCS for various Long COVID symptoms beyond fatigue.

By addressing these research priorities, we can move from theoretical potential to empirical validation of AI-guided tDCS as a treatment for Long COVID.

## 9. Global Implementation of AI-Guided tDCS for Long COVID

The implementation of AI-guided tDCS for Long COVID treatment presents unique challenges and opportunities across different healthcare systems worldwide. This section explores the considerations for global implementation, with a particular focus on resource-limited settings.

### 9.1. High-Income Countries (HICs)

In high-income countries, the primary challenges revolve around integration with existing healthcare infrastructure, navigating regulatory pathways, and developing sustainable reimbursement models.

#### 9.1.1. Integration with Existing Infrastructure

One of the key challenges in HICs is the seamless integration of AI-guided tDCS systems with existing healthcare infrastructure. This includes leveraging existing telemedicine platforms to incorporate AI-guided tDCS protocols. Torous et al. [76] discussed the potential of digital health technologies in psychiatry, providing insights that could be applied to the implementation of AI-guided tDCS for Long COVID. The authors emphasized the importance of interoperability and data standardization, which are crucial for integrating AI-guided tDCS systems with various electronic health record (EHR) systems.

For example, AI-guided tDCS systems could be designed to interface with existing EHR systems, allowing for automated data collection and the integration of treatment outcomes with patients’ overall health records. This integration could facilitate more comprehensive patient care and enable large-scale data analysis for the ongoing refinement of AI models.

#### 9.1.2. Regulatory Pathways

Navigating the regulatory landscape for AI-guided medical devices is another significant challenge in HICs. He et al. [77] explored the regulatory considerations for AI in medical devices, highlighting the need for adaptive regulatory frameworks that can keep pace with rapidly evolving AI technologies.

To address this challenge, researchers and developers should work closely with regulatory bodies such as the U.S. Food and Drug Administration (FDA) and the European Medicines Agency (EMA) to establish clear pathways for AI-guided medical devices. This might involve developing new regulatory categories that account for the adaptive nature of AI algorithms or creating frameworks for continuous post-market surveillance and updating of AI models.

Additionally, there is a need to develop standards for clinical validation of AI algorithms in tDCS applications. This could involve establishing benchmarks for algorithm performance, defining acceptable levels of explainability for clinical decision support systems, and creating protocols for ongoing monitoring and updating of AI models in clinical use.

#### 9.1.3. Reimbursement Models

Developing sustainable reimbursement models for AI-guided tDCS treatments is crucial for their widespread adoption in HICs. This involves engaging with insurance providers to develop appropriate coding and payment structures for these novel treatments.

Garrison et al. [78] discussed the challenges of value-based pricing for personalized medicine, providing insights that could be applied to AI-guided tDCS. The authors emphasized the need for innovative payment models that account for the value of personalized treatments and the potential for improved outcomes over time.

To support the development of these reimbursement models, it will be necessary to conduct robust cost-effectiveness studies. These studies should consider not only the direct costs of AI-guided tDCS treatment but also the potential long-term savings from improved outcomes and reduced healthcare utilization among Long COVID patients.

### 9.2. Middle-Income Countries (MICs)

In middle-income countries, the focus should be on adapting AI models to local contexts, building capacity for implementation, and leveraging public–private partnerships.

#### 9.2.1. Adaptation of AI Models

A key challenge in MICs is ensuring that AI models developed primarily in HICs are valid and effective in local populations. This requires validating and adapting AI models using local patient data to ensure relevance to the population. Kappen et al. [79] discussed the challenges of implementing AI in resource-constrained settings, emphasizing the importance of context-specific validation and adaptation of AI models.

Furthermore, developing multilingual interfaces is crucial to overcoming language barriers and ensuring the accessibility of AI-guided tDCS systems in diverse linguistic contexts. This might involve not only translating user interfaces but also adapting natural language processing models to local languages and dialects.

#### 9.2.2. Capacity Building

Investing in training programs for healthcare providers on the use of AI-guided tDCS systems is essential for successful implementation in MICs. This could involve developing comprehensive training curricula that cover both the technical aspects of tDCS administration and the interpretation of AI-generated treatment recommendations.

Collaborating with local universities to develop expertise in AI and neuromodulation is another important aspect of capacity building. Ding et al. [80] discussed strategies for building AI capacity in low- and middle-income countries, highlighting the importance of international collaborations and knowledge transfer programs.

#### 9.2.3. Public-Private Partnerships

Engaging with local tech companies to develop affordable AI-guided tDCS devices could help address cost barriers to implementation in MICs. This might involve adapting existing technologies to local manufacturing capabilities or developing novel, low-cost solutions tailored to the needs of resource-constrained settings.

Partnering with telecommunications companies to ensure reliable connectivity for telemedicine applications is also crucial, particularly for remote monitoring and adjustment of AI-guided tDCS treatments. Zhao et al. [81] explored the potential of 5 G technology in telemedicine, providing insights that could be applied to the implementation of AI-guided tDCS in MICs.

### 9.3. Low-Income Countries (LICs)

In low-income countries, the focus should be on developing simplified AI models, leveraging mobile health technologies, and implementing task-shifting strategies.

#### 9.3.1. Simplified AI Models

Developing “lite” versions of AI algorithms that can run on less powerful hardware is crucial for implementation in resource-constrained settings. This might involve focusing on essential features that provide the most clinical value, potentially sacrificing some degree of personalization or real-time adaptability in favor of broader accessibility. An alternative approach to these lite algorithms would be remote computing services, though this would require more careful anonymization in order to guarantee patient privacy. Wahl et al. [12] discussed the potential of AI in global health, emphasizing the need for context-appropriate AI solutions in low-resource settings. The authors highlighted the importance of developing AI models that can function effectively with limited computational resources and intermittent internet connectivity.

#### 9.3.2. Mobile Health (mHealth) Integration

Leveraging widespread mobile phone usage to develop smartphone-based interfaces for AI-guided tDCS systems could greatly enhance accessibility in LICs. This might involve creating mobile apps that can guide patients through home-based tDCS sessions, collect symptom data, and provide basic AI-driven treatment recommendations.

Implementing SMS-based symptom tracking and treatment adherence reminders could further enhance the effectiveness of AI-guided tDCS in settings where smartphone penetration is limited. Aranda-Jan et al. [82] reviewed the impact of mHealth projects in Africa, providing insights into the potential and challenges of mobile health technologies in low-resource settings.

#### 9.3.3. Task-Shifting Strategies

Training community health workers to administer AI-guided tDCS under remote supervision could help address workforce shortages in LICs. This approach could involve developing simplified training programs and user-friendly interfaces that allow non-specialist healthcare providers to safely administer tDCS treatments.

Developing AI-driven decision support tools to assist non-specialist healthcare providers in managing Long COVID patients could further enhance the effectiveness of task-shifting strategies. Schwalbe and Wahl [83] discussed the potential of AI-enabled task-shifting in global health, highlighting both the opportunities and ethical considerations of this approach.

### 9.4. Cross-Cutting Considerations

Several considerations are relevant across all economic contexts and are crucial for the successful global implementation of AI-guided tDCS for Long COVID.

#### 9.4.1. Affordability and Accessibility

Exploring tiered pricing models for AI-guided tDCS systems based on country income levels could help ensure global accessibility. This approach has been successfully implemented in other areas of healthcare, such as vaccine distribution [84]. For AI-guided tDCS, this might involve offering different versions of the technology with varying levels of functionality and cost, tailored to the resources and needs of different healthcare systems.

Developing open-source AI algorithms could significantly reduce licensing costs and promote innovation across diverse settings. Alqurana et al. [85] discussed the potential of open-source AI in healthcare, highlighting how it can foster collaboration and accelerate technological advancement, particularly in resource-limited settings.

#### 9.4.2. Cultural Adaptation

Ensuring AI models and interfaces are culturally appropriate and consider local health beliefs is crucial for patient acceptance and treatment adherence. This goes beyond mere translation and involves understanding and incorporating local conceptualizations of health, illness, and treatment.

Engaging with community leaders and patient groups to promote the acceptance of the technology is essential. Participatory design approaches, as described by Blandford et al. [86] in the context of digital health interventions, could be particularly valuable in ensuring that AI-guided tDCS systems are culturally acceptable and meet the needs of diverse populations.

#### 9.4.3. Data Privacy and Security

Implementing robust data protection measures that comply with local regulations is crucial, particularly given the sensitive nature of health data and the potential for AI models to handle large volumes of patient information. This might involve developing region-specific data handling protocols and encryption methods.

Exploring federated learning approaches could enable collaborative model development without centralizing sensitive patient data. This approach, as described by Rieke et al. [51], allows AI models to be trained on decentralized data, potentially addressing both privacy concerns and the challenge of data scarcity in some settings.

#### 9.4.4. Sustainable Implementation

Developing solar-powered or long-lasting battery options for tDCS devices could address the challenge of unreliable electricity in many low- and middle-income countries. Pillai et al. [87] discussed the potential of solar-powered medical devices in resource-limited settings, providing insights that could be applied to the design of AI-guided tDCS systems.

Creating modular systems that allow for easy maintenance and upgrades is crucial for long-term sustainability. This approach, often used in frugal innovation [88], could enable local technicians to repair and update AI-guided tDCS systems, reducing the dependence on external support.

#### 9.4.5. Global Collaboration and Knowledge Sharing

Establishing international research consortia to share data and best practices could accelerate the global implementation of AI-guided tDCS for Long COVID. Such collaborations could facilitate the development of more robust and generalizable AI models, as well as the sharing of implementation strategies across diverse settings.

Developing global registries to track long-term outcomes and safety across diverse populations is crucial for the ongoing refinement and validation of AI-guided tDCS approaches. Wilkinson et al. [89] discussed the importance of data sharing in global health research, principles that could be applied to the development of such registries for AI-guided tDCS in Long COVID.

### 9.5. Challenges and Future Directions

#### 9.5.1. Digital Divide

Addressing disparities in internet access and digital literacy that may limit the reach of AI-guided tDCS remains a significant challenge. Strategies to bridge this divide, as discussed by Abebaw et al. [90] in the context of digital health in Africa, could be adapted for AI-guided tDCS implementation.

Developing offline capabilities for AI algorithms to function in areas with limited connectivity is crucial. This might involve creating AI models that can run locally on low-power devices, with periodic updates when connectivity is available.

#### 9.5.2. Workforce Development

Investing in education and training programs to build local capacity in AI and neuromodulation is essential for sustainable implementation. This could involve partnerships with local universities, online learning platforms, and international knowledge exchange programs.

Creating international exchange programs to facilitate knowledge transfer between HICs and LMICs could accelerate capacity building. Such programs could be modeled on successful initiatives in other areas of global health, as described by Ouma and Dimaras [91].

#### 9.5.3. Ethical Considerations

Developing global ethical guidelines for AI-guided neuromodulation that respect cultural differences is crucial. This process should involve diverse stakeholders and consider varying cultural perspectives on autonomy, privacy, and the role of technology in healthcare.

Ensuring equitable access to AI-guided tDCS technologies to prevent the exacerbation of global health disparities remains a key ethical challenge. Addressing this may require innovative financing models, technology transfer agreements, and global cooperation.

#### 9.5.4. Long-Term Sustainability

Developing business models that ensure the long-term viability of AI-guided tDCS programs in resource-limited settings is crucial. This might involve exploring social enterprise models, public–private partnerships, or integration with existing health system financing mechanisms.

Encouraging local innovation and manufacturing to reduce the dependence on imported technologies could enhance sustainability and promote economic development. Initiatives to support local medical device manufacturing, such as those described by Arasaratnam and Humphreys [92], could be adapted to support the production of AI-guided tDCS systems.

In conclusion, the global implementation of AI-guided tDCS for Long COVID treatment requires careful consideration of diverse healthcare contexts, innovative approaches to technology adaptation and delivery, and a commitment to equity and sustainability. By addressing these challenges and leveraging opportunities for collaboration and knowledge sharing, AI-guided tDCS has the potential to significantly impact Long COVID care on a global scale.

##### Limitations and Risks of AI in tDCS for Long COVID Treatment

While this article has explored the potential benefits of AI-guided tDCS for Long COVID treatment, it is crucial to acknowledge the current limitations of AI technology and the potential risks associated with its implementation in this context. This section aims to provide a balanced perspective on the capabilities of AI and the challenges that need to be addressed.

##### Current Limitations of AI in Healthcare

1. Data Quality and Quantity: AI models are only as good as the data they are trained on. In the context of Long COVID, which is a relatively new condition, there may be insufficient high-quality data available for training robust AI models [54].
2. Generalizability: AI models developed using data from specific populations may not generalize well to other groups, potentially leading to biased or inaccurate recommendations for certain patients [93].
3. Interpretability: Many advanced AI models, particularly deep learning models, operate as “black boxes”, making it difficult to understand how they arrive at their decisions. This lack of interpretability can be problematic in healthcare settings where clinicians need to understand and explain treatment decisions [54].
4. Adaptability to New Information: While AI can process large amounts of data, it may struggle to quickly adapt to new information or changing circumstances, which is particularly relevant in the rapidly evolving field of Long COVID research [94].

##### Potential Risks and Challenges

1. Overreliance on AI: There is a risk that clinicians might overly rely on AI recommendations, potentially overlooking important clinical judgments that fall outside AI’s parameters [95].
2. Privacy and Security Concerns: The use of AI in healthcare involves processing large amounts of sensitive patient data, raising concerns about data privacy and security [96].
3. Regulatory Hurdles: The rapidly evolving nature of AI technology presents challenges for regulatory bodies in ensuring safety and efficacy while not stifling innovation [97].
4. Ethical Considerations: The use of AI in clinical decision-making raises ethical questions about accountability, consent, and the potential for AI to exacerbate existing healthcare disparities [11].
5. Integration with Existing Clinical Workflows: Implementing AI-guided tDCS systems may require significant changes to existing clinical practices, which can be met with resistance and may introduce new sources of error if not carefully managed [70].

##### Balancing Potential with Realism

While AI holds promise for optimizing tDCS protocols and potentially improving outcomes for Long COVID patients, it is important to approach its implementation with cautious optimism. Current AI capabilities, while impressive, are not without limitations:

1. AI cannot replace clinical expertise: AI should be viewed as a tool to augment clinical decision-making, not replace it. The complex and often unpredictable nature of Long COVID requires the nuanced understanding that experienced clinicians provide [77].
2. Continuous evaluation is necessary: As AI systems are implemented, their performance should be continuously monitored and evaluated against standard care to ensure they are truly improving patient outcomes [98].
3. Transparent communication is crucial: Patients should be clearly informed about the role of AI in their treatment, including its limitations and the extent of its influence on clinical decisions [99].
4. Multidisciplinary collaboration is key: The successful implementation of AI-guided tDCS will require close collaboration between clinicians, AI experts, ethicists, and regulatory bodies to navigate the complex landscape of clinical AI [100].

By acknowledging these limitations and potential risks, we can work towards responsible development and implementation of AI-guided tDCS for Long COVID treatment. This approach allows us to harness the potential benefits of AI while maintaining a realistic view of its current capabilities and the challenges that lie ahead.

##### Practical Implementation Steps for AI-Guided tDCS in Clinical Settings

While the potential of AI-guided tDCS for Long COVID treatment is promising, translating this technology into clinical practice requires careful planning and execution. This section outlines specific steps for implementing AI-guided tDCS in real clinical settings, addressing the complexity of the process and potential challenges.

1. Infrastructure Setup
  (a) Hardware Requirements:
    Acquire FDA-approved tDCS devices capable of remote parameter adjustment.
    Ensure secure, high-speed internet connectivity in clinical areas.
    Set up secure servers for data storage and AI model hosting.
  (b) Software Development:
    Develop user-friendly interfaces for clinicians and patients.
    Create secure data pipelines for real-time data collection and processing.
    Implement AI models with appropriate security measures.
  (c) Integration with Existing Systems:
    Establish interoperability with Electronic Health Record (EHR) systems.
    Ensure compatibility with existing clinical workflow management tools.
2. AI Model Development and Validation
  (a) Data Collection:
    Establish protocols for standardized data collection across multiple sites.
    Implement privacy-preserving techniques such as federated learning [6].
  (b) Model Development:
    Develop and train AI models using historical tDCS data and Long COVID patient information.
    Employ techniques like transfer learning to adapt existing models to Long COVID specifics.
  (c) Clinical Validation:
    Conduct pilot studies to validate AI model predictions against expert clinician decisions.
    Perform iterative refinement based on clinical feedback and outcomes.
3. Clinical Protocol Development
  (a) Treatment Guidelines:
    Develop clear guidelines for patient selection and exclusion criteria.
    Establish protocols for baseline assessments and ongoing monitoring.
  (b) Safety Protocols:
    Implement real-time safety monitoring systems.
    Develop clear procedures for managing adverse events.
  (c) Clinician Training:
    Create comprehensive training programs for clinicians on AI-guided tDCS use.
    Develop resources for ongoing support and troubleshooting.
4. Regulatory Compliance and Approval
  (a) FDA Approval:
    Engage with the FDA early to understand requirements for AI-guided medical devices [101].
    Conduct necessary clinical trials to demonstrate safety and efficacy.
  (b) Data Protection:
    Ensure compliance with HIPAA and other relevant data protection regulations.
    Implement robust data anonymization and encryption protocols.
  (c) Ethical Review: Obtain approval from institutional review boards (IRBs) for clinical implementation
    Address ethical considerations related to AI decision-making in healthcare.
5. Clinical Integration
  (a) Pilot Implementation:
    Start with a small-scale pilot in select clinics.
    Monitor closely and gather feedback from clinicians and patients.
  (b) Workflow Integration:
    Develop standard operating procedures (SOPs) for integrating AI-guided tDCS into daily clinical practice.
    Create decision support tools to assist clinicians in interpreting AI recommendations.
  (c) Quality Assurance:
    Implement continuous monitoring systems to track treatment outcomes.
    Establish regular review processes to assess and improve system performance.
6. Scaling and Maintenance
  (a) Gradual Expansion:
    Develop a phased roll-out plan to expand to more clinical sites.
    Provide adequate support and resources for each expansion phase.
  (b) Ongoing Model Updates:
    Establish protocols for regular model updates based on new data.
    Implement a system for version control and tracking of AI model changes.
  (c) Long-term Monitoring:
    Set up systems for long-term efficacy and safety monitoring.
    Establish channels for ongoing feedback from clinicians and patients.
7. Education and Communication
  (a) Patient Education:
    Develop materials to educate patients about AI-guided tDCS.
    Create informed consent processes that clearly explain the role of AI in treatment.
  (b) Public Engagement:
    Engage in transparent communication about the use of AI in healthcare.
    Address potential concerns and misconceptions about AI-guided treatments.
  (c) Professional Development:
    Organize workshops and conferences for knowledge sharing among clinicians.
    Collaborate with medical schools to incorporate AI-guided neuromodulation into curricula.

By following these steps, healthcare providers can navigate the complexities of implementing AI-guided tDCS in clinical settings. It is important to note that this process requires a multidisciplinary approach, involving clinicians, AI experts, biomedical engineers, and regulatory specialists. Additionally, the implementation should be viewed as an iterative process, with continuous refinement based on clinical experience and emerging research.

## 10. Conclusions: The Future of AI-Guided tDCS for Long COVID

AI-guided transcranial direct current stimulation (tDCS) for Long COVID treatment represents a promising frontier in medical technology, integrating neuroscience, artificial intelligence, and personalized medicine. This approach offers the potential for enhanced treatment efficacy through personalized stimulation parameters and adaptive protocols. AI applications in clinical trial design could accelerate research progress, addressing the unique challenges posed by Long COVID’s variability. While current empirical evidence supporting AI-guided tDCS for Long COVID is limited, insights from related fields provide a foundation for our hypotheses and highlight key areas for future investigation. The implementation of AI-guided tDCS in clinical settings presents complex challenges, and a structured approach involving careful planning, multidisciplinary collaboration, and iterative refinement can pave the way for successful integration of this promising technology in Long COVID treatment.

However, significant challenges remain. Ethical considerations, including data privacy, equitable access, and AI transparency, must be carefully addressed. The efficacy of tDCS for Long COVID symptoms remains uncertain, and AI systems may struggle to generalize across diverse populations. Regulatory hurdles, long-term effects of treatment, and potential data biases require thorough investigation.

Global implementation presents varying challenges across different economic contexts, necessitating context-specific solutions. Key themes for future development include interdisciplinary collaboration, personalization, ethical innovation, global equity, patient-centered approaches, and continuous learning systems.

Moving forward, balancing innovation with caution is crucial. While significant work remains, AI-guided tDCS holds the potential to improve the lives of those affected by Long COVID. Through careful navigation of technical, ethical, and practical challenges, this approach could contribute significantly to Long COVID management and broader neuromodulation applications.

Continued research, ethical vigilance, and a commitment to global equity will be essential in realizing the potential of AI-guided tDCS, potentially transforming our approach to Long COVID treatment and expanding our understanding of personalized neuromodulation therapies.

## Figures and Tables

**Table 1:** Overview of various AI techniques and how they might be applied specifically to optimizing tDCS for Long COVID treatment.

AI Technique	Description	Potential Applications in tDCS for Long COVID
Support Vector Machines (SVM)	Supervised learning model for classification and regression	- Predicting patient response to specific tDCS protocols - Classifying patients into subgroups based on symptom profiles
Random Forests	Ensemble learning method using multiple decision trees	- Identifying important features for tDCS response prediction - Handling complex interactions between patient characteristics and tDCS parameters
Deep Learning (Neural Networks)	Multi-layer neural networks capable of learning complex patterns for classification and regression	- Analyzing multi-modal data (clinical, neuroimaging, physiological) - Real-time optimization of tDCS parameters based on ongoing patient responses
Reinforcement Learning	Learning optimal actions through trial and error	- Dynamically adjusting tDCS parameters over multiple sessions - Optimizing long-term treatment strategies
Natural Language Processing (NLP)	Processing and analyzing human language	- Analyzing patient-reported outcomes and symptom descriptions - Extracting relevant information from medical records for tDCS optimization
Clustering Algorithms (e.g., K-means)	Unsupervised learning for identifying groups in data	- Discovering patient subgroups with similar tDCS response patterns - Identifying common symptom clusters in Long COVID for targeted tDCS protocols
Bayesian Optimization	Efficient optimization of black-box functions	- Efficiently searching the parameter space for optimal tDCS settings - Balancing exploration and exploitation in clinical trials

**Table 2:** Future research directions for AI-guided tDCS in Long COVID treatment.

Research Area	Key Objectives	Potential Approaches
1. Optimal tDCS Parameters	Determine effective montages and stimulation parameters for different Long COVID symptoms	Large-scale, randomized controlled trials with AI analysis of patient characteristics and outcomes
2. AI-driven Personalization	Develop AI algorithms to predict individual patient responses and enable real-time parameter adjustment	Machine learning models using large datasets of patient characteristics and tDCS outcomes; Closed-loop tDCS systems with AI-driven parameter adjustment
3. Long-term Efficacy and Safety	Understand long-term effects and potential cumulative effects or safety concerns	Longitudinal studies (12–24 months) with AI analysis of long-term outcome patterns and safety signals
4. Integration with Other Treatments	Investigate interactions between AI-guided tDCS and other Long COVID treatments	Factorial studies combining tDCS with other treatments, using AI to analyze interaction effects
5. Neuroplasticity and Mechanism of Action	Investigate how AI-guided tDCS affects brain plasticity and connectivity	Studies combining tDCS with neuroimaging (fMRI, EEG) and AI analysis of brain changes
6. Combining tDCS with Neuroimaging	Develop imaging-based biomarkers for predicting and monitoring tDCS response	Multimodal imaging approaches (PET, fMRI, EEG) with AI analysis of complex datasets
7. Patient-Reported Outcomes and Quality of Life	Incorporate patient-centered outcomes into AI models for tDCS optimization	AI models integrating standardized quality of life measures and free-text patient narratives
8. Home-Based AI-Guided tDCS	Investigate feasibility and safety of home-based, AI-guided tDCS	Feasibility studies of home-based tDCS systems with AI monitoring
9. Health Economic Analysis	Understand cost-effectiveness of AI-guided tDCS compared to standard care	Health economic modeling studies incorporating treatment efficacy, resource utilization, and quality of life data
10. Ethical and Regulatory Frameworks	Develop comprehensive ethical guidelines and regulatory frameworks	Multidisciplinary studies involving ethicists, legal experts, clinicians, and patients
11. Generalizability and Health Disparities	Investigate generalizability of AI models across populations and potential to address health disparities	Multi-center, international studies; Targeted studies in underserved communities

**Table 3:** Integration of neuroimaging modalities with AI-guided tDCS.

Neuroimaging Modality	Data Provided	AI Application	Potential Impact on tDCS
PET (Positron Emission Tomography)	Metabolic activity, Neuroinflammation	Pattern recognition for identifying hyperactive/hypoactive regions	Target selection for tDCS montage
fMRI (functional Magnetic Resonance Imaging)	Brain activation patterns, Functional connectivity	Network analysis, Dynamic causal modeling	Optimization of stimulation parameters based on functional connectivity
sMRI (structural MRI)	Brain structure, White matter integrity	Voxel-based morphometry, Tract-based spatial statistics	Personalization of electrode placement based on individual anatomy
DTI (Diffusion Tensor Imaging)	White matter tractography	Graph theoretical analysis of structural connectome	Targeting of specific white matter tracts for stimulation
EEG (Electroencephalography)	Cortical oscillations, Event-related potentials	Machine learning for EEG feature extraction, Real-time signal processing	Closed-loop tDCS with adaptive stimulation based on ongoing brain activity
MEG (Magnetoencephalography)	Source-localized neural activity	AI-driven source reconstruction, Functional connectivity analysis	Precise targeting of deep brain regions for stimulation
